# Acknowledgment to Reviewers of *Toxins* in 2021

**DOI:** 10.3390/toxins14020106

**Published:** 2022-01-30

**Authors:** Toxins Editorial Office

**Affiliations:** MDPI AG, St. Alban-Anlage 66, 4052 Basel, Switzerland

Rigorous peer-reviews are the basis of high-quality academic publishing. Thanks to the great efforts of our reviewers, *Toxins* was able to maintain its standards for the high quality of its published papers. Thanks to the contribution of our reviewers, in 2021, the median time to first decision was 17 days and the median time to publication was 37 days. The editors would like to extend their gratitude and recognition to the following reviewers for their precious time and dedication, regardless of whether the papers they reviewed were finally published:
Aaron FullerJun KameiAaron M. NeimanJunseo OhAaron MayJussi MeriluotoAbdolrahman KhezriJustin RenaudAbdul QayyumJustin SchmidtAbolfazl JahangirlJustina PradaAdam LiwoJustyna Lalak-KańczugowskaAdam OkorskiJyh-Cherng JuAdam ReitzelJyunichiro James KazamaAdam SchrumKai ZhangAdela Lopez De CerainKaitlyn BissonnetteAdela R. Mauri-AucejoKaiwen ChuangAdnan Shami ShahKalle OlliAdnan ŠišićKalyan K. DewanAdolfo PerrottaKam-Chau WuAdrian WaggKamil JastrzębskiAdrijana LeonardiKanniah RajasekaranAdva MechalyKarel EveraertAgata FabiszewskaKarina Van Der LindeAgata KaczmarekKarla De Castro Figueiredo BordonAgnieszka BossowskaKarolina ChilickaAgnieszka Piastowska-CiesielskaKarolina GromadzkaAgustín AriñoKarolina HlavováAhmad AlshannaqKatarzyna GolanAhmed BadrKatarzyna MarchwińskaAhmed HasanKatarzyna PacygaAibo WuKatarzyna SzałapataAisha KhatoonKateřina BreiterováAitor RementeriaKateřina DadákováAjit K. BasakKatharina ErnstAksel BernhoftKathleen HefferonAkshay ModiKathleen WalterAlastair HutchisonKatia CailliauAlberto Rico YusteKatja KolleweAldo LaganàKatrin SchäferAldo NicosiaKatrin TeubnerAlejandro Martin-GorgojoKazuya MurataAleksandar ShkondrovKeenan MintzAleksandra SzydłowskaKeerthi S. GurugeAlessandra DubbiniKeiichi SumidaAlessandra PalladiniKeiji SugiuraAlexander DilmanKeith WarrinerAlexander HawlitschkaKeith WeaverAlexander MuellerKen TeterAlexandra MalachováKen WinkelAlexandre KreislerKenneth A. CallicottAlexandre LamasKenneth J. GenoveseAlican GulsevinKent M. ReedAlicia BoltKera E. LuckritzAlicia RodríguezKerr GrahamAlicja SułekKeshav GopalAlix PierronKevin FitzsimmonsAlja ŠternKevin F.M. ReedAllan Rod MerrillKevin WelchAlma Vázquez-DuránKhaled Mohammed GebaAmanda PacholakKhianoush Khosravi-DaraniAmanda S. KorenoskiKhushwant Singh BhullarAmandine CaruanaKichul ChoAmber C. BeckettKinga MusiałAmelia BrunaniKinga Stuper-SzablewskaAmin Mousavi KhaneghahKiriake AyaAmin SayyariKirill AntonetsAmina RhouatiKiyotaka NishikawaAmir IsmailKlemen ČandekAmir Mohammad MalvandiKo-Hua TsoAmira AbugomaaKonrad A. SzychowskiAmit V. PandeyKonstantin ChekanovAmparo AlfonsoKonstantina PyrgakiAna Da SilvaKonstantinos VavitsasAna GuimarãesKristin M. SchillAna I. Lopez NavasKristina MastanjevićAna Leonor Abrahão NencioniKristina MenaAna Luisa PereiraKrzysztof JuśAna Piedra-BuenaKwang-Soo ShinAna-Rosa BallesterKyu-Ho YiAnas CherquiKyungjae-andrew YoonAndré BillionLaetitia DouAndré FulyLama NazzalAndré Lopes FulyLambertus Van Den HeuvelAndrea DidierLander Egana GorronoAndrea GiansantiLara ManyesAndrea KunovaLarelle FabbroAndrea PulvirentiLasse LindahlAndreas AltenburgerLaura BiessyAndreas H. LaustenLaura Carbonell-RozasAndreas SegerLaura EscriváAndreja KustLaura Miller ConradAndrew C. PoveyLaura Moreno MesoneroAndrew PadulaLaura RighettiAndrew PoveyLaura-Oana AlbulescuAndrew WalkerLaurence Meslet-CladiereAndrias O'ReillyLaurent ChiarelliAndrzej KrezelLaurent MetzingerAndrzej P. HermanLaurentiu Mihai PaladeAndy PickettLeao Martins José ManuelAngela MallyLeda GiannuzziAngela Melton-CelsaLekha SlenoAngela MontoneLene Heise GarveyAngelo RomanoLenka ŠindlerováAngels TudóLeonardo CerasinoAnie Priscilla MasilamaniLeonor MeiselAnisha Mahendra ThankiLeticia Díez-QuijadaAnita MikolajczykLiang ChenAnja Van CampenhoutLicia PantanoAnna Baturo-CiesniewskaLidia ChomiczAnna DragošLi-Han HsuAnna Katarzyna WronskaLikwang ChenAnna M. Kurek-GóreckaLiliana J.G. SilvaAnna Maria AloisiLinda BöswaldAnna PawlikLinda HarrisAnna ShepelyakovskayaLing-Wen DingAnna SieroslawskaLinlin MaAnna StarzyńskaLise Nistrup JørgensenAnna Szczerba-TurekLiselott Svensson StadlerAnna ZaghiniLoïc CoutteAnnabel BirueteLorella SeverinoAnnageldi TayyrovLorena Durán-RiverollAnnamaria GerardinoLouise R. PageAnnamaria MincuzziLoukia EkateriniadouAnnarita FalangaLourival PossaniAnne E. BoyerLuc IngenbleekAnthi RanellaLuca TirloniAnthony WenndtLucas LeclèreAntía Lestido-CardamaLucia GambacortaAntimo Di MaroLucía García-OrtegaAnton E. ShikovLucia Helena FaccioliAntônio Domingos BrescovitLucía SoliñoAntonio GuerrieriLuciana TartaglioneAntonio PortugalLuigi Alberto PiniAnupam Datta GuptaLuigi La PietraAraceli Escudeiro RossignoliLuigi MacchiaArakere Chunchegowda UdayashankarLuigi SantacroceAram MegighianLuís AbrunhosaArantza VitoriaLuís Gustavo Romani FernandesAravind ThavamaniLuís M. FélixAriadna BargielaLuis Roberto De Camargo GonçalvesArianna Di StadioLuis Sánchez-del-CampoArianna PiersantiŁukasz StępieńArif SiddiquiŁukasz TomczykArijit NathŁukasz ZielonkaArin MarchesiŁukasz ŻielonkaArmah A. De La CruzLyudmila DimitrovaArmel GalletM. Carmen Louzao OjedaArtem ErmakovMaarten De MolArturo AnadónMaciej BuśkoAshutosh BahugunaMaciej GagatAsparouh I. IlievMaciej GajęckiAthanasios AlegakisMaciej LechAtmika PaudelMaciej SkorackiAtsushi KoikeMadhu KamleAtsushi TogawaMaëlle JaouannetAudrey Roy-LachapelleMagali CasanovaAurelian-Sorin PașcaMagdalena BarešováAwdhesh MishraMagdalena Buszewska-ForajtaAziz KarakayaMagdalena GajęckaBania JacekMagdalena Polak-ŚliwińskaBarbara BlackwellMagdalena SobieskaBarbara C. KahlMagdalena ToporowskaBarbara Illowsky KarpMagdi El FerganiBarbara Pawlik-SkowrońkaMahmoud F. SeleimanBarbara Przybylska-GornowiczMaia PeraicaBarry RosenMaike KrauseBartłomiej GrygorcewiczMaja Jazvinšćak JembrekBasheer IqdiamMaja Šegvić KlarićBeata MessyaszMałgorzata AdamczukBeelee ChuaMałgorzata BonisławskaBehrouz ShamsaeIMalgorzata OlejnikBelén PatiñoMałgorzata PiotrowskaBen GreenMałgorzata ŚwiątkiewiczBenedikt CramerMałgorzata SztankeBenedito C. PrezotoMamatha BallalBenjamin JurekManabu AsakawaBenjamin MaskreyMandar JogBenjamin-Florian HempelManel LeiraBerenice Y. GitomerManoella SibatBernadette TelekyMarc L. De BuyzereBernard PoulainMarc MarescaBernd RodemannMarcel SchneiderBettina HoeverMarcelo FerreiraBettina SeegerMarcin BryłaBiancamaria CiascaMarcin RóżewiczBieito Fernandez CastroMárcio José De Abreu RodriguesBiing Hui LiuMarco GerdolBin WangMarco MasiBiresaw AberaMarco PirazziniBirgit M. PruessMarco RomanoBishwo N. AdhikariMarco SaracchiBjörn MeijersMarek KalenikBlair W. PerryMarek KieliszekBogdan Gabriel ȘlencuMarek RenčoBogusław BuszewskiMarek WiergowskiBogusław OlkowskiMargarita FernandezBojan KnapMargot ErnstBojan ŠarkanMari Yotsu-YamashitaBradley A. SpicerMaria A. AnderssonBradley HernlemMaria Alice Da Cruz-HöflingBradley L. UrquhartMaría Ángeles LezcanoBrady R. CunninghamMaria Angeles RojoBranimir K. HackenbergerMaria BelousovaBreda Jakovac-StrajnMaría Blanco-DiazBrett R. HamiltonMaria CappuccilliBrian BermanMaría Del Pilar SuárezBrigitte G. DornerMaria Del Rosario IglesiasBrigitte GirardMaria Francesca PeruzyBrittany HarlowMaría García PortelaBrittany N. SprecherMaria GeorgiadouBruno Ramos-MolinaMaria Ilenia De BartoloBruno RizzutiMaría J. AndradeBryan StegelmeierMaria Joao BotelhoByoung Sik KimMaria KazimírováByung Rae JinMaria Luiza Vilela OlivaC. Benjamin NamanMaría Luz Pérez-Parallé MeraC. Elizabeth StokesMaria Madalena SobralCalogero Di BellaMaría Moreno-del ÁlamoCameron B. GundersenMaria Rambla-AlegreCarl Mikael ÖbergMaria SachkovaCarlamaria ZojaMaría Soledad Sacristán BenayasCarlo CampobassoMaria ZoumpaniotiCarlo D'AscenziMaria-Fernanda Lorenzo-GómezCarlo Ettore PellicciariMaria-Giuliana VannucchiCarlos Alfredo Gómez BravoMarialessandra ContinoCarlos BicudoMariam HassanCarlos Correa-NettoMarianna RanieriCarlos M. MartínezMarianthi BasalekouCarlotta BalconiMarie-Helene MarionCarmela Maria MontoneMarilena GiliCarmelo ChisariMarina AboalCarmen BarcenaMarinônio Lopes CornélioCarmen FerrerMario FernándezCarmen LiMario MasielloCarmen SolcanMario Segio PalmaCarmen ValeMarion EhrichCarol TrimMarisa MegnaCarolina LúquezMarius ParaschivCaroline FabiouxMariya KiselevaCaroline Le MaréchalMark EstienneCaroline S. MurphyMark McClainCaroline StrubMark WeaverCarsten HolzmannMarko HaloCarter MitchellMarkus KranzlerCassandra ModahlMarkus NaumannCătălin MaximMaroula G. KokotouCaterina MorciaMarsha MorganCatherine Dunyach-RemyMarta Anna KowalikCecilia PozziMarta BełkaCecilie KnudsenMárta ErdélyiCedric ChaverouxMarta Garcia-ClementeCees WaalwijkMarta HerreraCeline MeerpoelMarta Pastor-BeldaCésar MatteiMartha M. EiblCesare AccinelliMartha Marie VaughanCezary TkaczukMartin PospíšekChandrashekhar Devidas PatilMartin SchmelzChanglong ShuMartina OchsChang-Yi WuMartina PianoChao LiuMartina ŠtamparCharles JoussainMartina TorricelliCharles M. MacVeanMasachika NiimiCharng-Cherng ChyauMasahiro EriguchiCherie F. GambleyMasahiro HiramaChia-Chi HuangMasahiro OgawaChiara BarisioneMasataka OdaChiara Dall'AstaMasayo KushiroChiara FerraciniMasayori InouyeChia-Yang LiMasayuki SatakeChi-chung ChouMassimiliano CuccioloniChien-Te LeeMassimo D’AgostinoChih-Chin YangMassimo PicardoChi-Kong ChanMateja Manček-KeberChing-Yao HuMateusz Jakub DąsalChit Shing Jackson WooMathilde MontibusChitrita DebRoyMatt LewinChris MaragosMatteo A. SurdoChristian LegrosMatteo Della PortaChristian MüllerMatthew A. RobertsChristian RüterMatthew BeardChristian WöberMatthew BeckChristina C. TamMatthew HoldingChristina Nielsen-LeRouxMatthew LebarChristina SchroederMatthew LewinChristine CoustauMatthew Q. MillerChristine EdwardsMatthew TaylorChristine Rasetti-EscargueilMatthias BrennerChristof AignerMatthias KochChristoph BueschlMatthias WalterChristopher J. SilvaMaurizio BrigottiChristopher William HollandMauro Cesar Palmeira VilarChristos GatsogiannisMaxim BurkinChuan Chiang-NiMaya AzradChueh-Hung WuMazen SalehChu-Fang LoMd Abdul HakimChul-Su YangMd Ashik UllahChung-Cheng WangMédéric LoyezClaudia BusoneroMeiji Soe AungClaudia DurantiMelinda FogarasiClaudia HessMeng LuoClaudia WiegandMeng-Yu WuCláudio ParenteMi Young LimClifton FagerquistMichael AppellColin BerryMichael DomingueConstanze PietschMichael E. OlsonCraig WilkieMichael FasulloCristina DaiaMichael G. WellerCristina Juan GarcíaMichael H.B. StowellCsilla PelyheMichal BogusiewiczCynthia Adaku ChilakaMichal CeremugaDaan KamphuisMichał Da̧browskiDabor ResiereMichal LetekDaiana Silva LopesMichał PiegzaDalal Hammoudi HalatMichał ZłochDamian PiotrowskiMichel DugonDaniel A. PowellMichel TreilhouDaniel C. NelsonMichela MitchellDaniel Echeverria-EsnalMichelle S. MostromDaniel FologeaMickaël LafondDaniel GilletMie KristensenDaniel HesselsonMiguel A. PrietoDaniel KamińskiMiguel MatillaDaniel KlichMi-Heon RyuDaniel L. GalvanMike ArmourDaniel SternMikhail MartchenkoDaniela Cajado-CarvalhoMiklós MézesDaniela JakšićMiklós PoórDaniele CastellaniMilena JankowskaDanielle Duanis-AssafMilva PepiDanijela Horvatek TomicMin WangDanúbia Da Cunha De Sá-CaputoMing Feng LiaoDany Domínguez-PérezMingjing SunDariusz DzigaMing-Yi ShenDavid A. DavisMin-Jung KangDavid A. GronebergMiran BrvarDavid BoelsMiriam HaidukowskiDavid Hernández HerreroMirja Salkinoja-SalonenDavid HerrinMirko CucinaDavid NelsenMiroslav CaboňDavid OwensMiroslav JuráčekDavide LonatiMitchell R. McGillDejan JakimovskiMitja KosnikDenis PiérardMohamed Al-FatimiDennis NurjadiMohamed F. AbdallahDerry K. MercerMohamed Fathi AbdallahDevavrat NeneMohammad AlimohammadiDianzhi HouMohanraj SadasivamDiego MartinezMonia LenziDimitra J. DafereraMonica Cartelle GestalDimitra TsounidiMonika Dhanji-RapkovaDimitrios P. PapachristosMonika JaneczkoDimitrios VarvaroussisMonika SzajwajDimitris A. GougoulisMonika WasilewskaDina Rešetar MaslovMontamas SuntravatDipak KathayatMoo-Seung LeeDirk Van HeldenMorana JaganjacDmitry AmininMostafa Abdel-GlilDmitry EnikeevMuhittin Cenk AkbostanciDomenico Azarnia TehranMunazza KiranDomenico DavolosMyriam BormansDomenico IntisoMyriam CuadradoDominic C. JennerMyung-Bok WieDominik DłuskiNadezhda Yuilevna StepanovaDominique M. VotionNadja HellmannDongying LiNancy SpanòDonn JohnsonNanda Kishore RouthuDörthe BrüggmannNanqin GanDuarte DiazNaoki IkegayaDuc Cuong BuiNaomasa OshiroĐuro JosićNatalia CampilloDyana OdehNatalia SkiepkoEdgar CambazaNataša HojnikEdoardo RaposioNaveed MalekEdvin PozharskiyNegisa Seyed ToutounchiEdward SionovNermeen YosriEiichi KumamotoNevenka ĆelepirovićEikan MishimaNicholas CarbonettiEkaterina M. NestorovichNicholas H. CarbonettiEkaterina N. LyukmanovaNicki NiemannElda DervishiNicola LandiElena B. AverinaNicola OreficeElena CircellaNicolas DelcourtElena EfremenkoNicolas PersonnicElena RampanelliNikola HodkovicováEleonora MarianNikola Zlatkov KolevEliana L. Faquim-MauroNilay Köse-VogelEliane Candiani BragaNina KudumijaElias AlisaacNkemdinma Uche-OkereaforElisabete ValérioNobumichi KobayashiElisabeth VargaNolwenn HymeryElisabetta CapraiNoor Azira Abd MutalibEliza MatuszewskaNóra AdányiElizabeth A. LeadbetterNoritaka SaekiElizabeth I. HamelinNorival Santos-FilhoElliott J. WrightNoureddine BouaïchaEloísa SevillaNourolah SoltaniElsa BonnaféNúria Sempere-RubioElsa T. RodriguesOfir SchusterElżbieta PatkowskaOier EtxebesteEmanuela GregoriOksana V NekrasovaEmelyn SalazarOlga A. GoryachevaEmily G. GreengardOlga A. KoksharovaEmily RalstonOlga A. SinitsynaEmmanouil KokkinakisOlga AvrutinaEmmanuel K. TangniOlga BelykhEnric ReverterOlga D. LopinaEnrico SangiovanniOlga KovbasnjukEran BosisOlga Lepšová-SkácelováErasmia SidiropoulouOlga OrlovaEric AbadieOlga P. GavrilovaEric D. MerkleyOlivier MicheauEric GontierOlivier PuelEric JohnsonOlubukola O. AjigboyeErica GiarratanoOluwatobi KolawoleErica J. MartiOmer BardaErica PackOndrej AdamovskyErik CerratoOrly Laskar-LevyErlinda TheOrna Staretz-ChachamErwin MärtlbauerOrnella RossettoEsperanza Rivera-De-TorreOsamu ArakawaEstefanía RodríguezOsbaldo Lopez-CharcasEstelle MarionOsmindo Rodrigues Pires JúniorEsther NistalOsnat RosenEszter Fliszár-NyúlOualid TalhiEugénia De AndradePablo Estevez BastosEustachio TarascoPablo HerreroEva AlonsoPalanisamy SubramanianÉva KorposPanagiota KatikouEva M. MateoPanagiota XaplanteriEva Mischak WeissingerPanagiotis SapountzisEvelien SnauwaertPantelis NatskoulisEwa LepiarczykPaola BadinoEwa TomaszewskaPaola GiorniEwelina KowalczykPaola VenierF. Matthew KuhlmannPaolo PolidoriFabian MuellerParamanandham KrishnamoorthyFabien DarfeuilleParisa GazeraniFabrizio AnniballiPaschalitsa TryfinopoulouFabrizio RuecaPatricia TesterFahimeh CharbgooPatrick Michael McNuttFarhad HossainPatrick RockenfellerFatemeh Azadegan-DehkordiPatrick WeydtFatima El KhalloufiPatrycja KleczkowskaFederico Maria RubinoPaul D’AgostinoFederico NalessoPaul M. D’AgostinoFelice MastroleoPaul SicilianoFelipe Penagos-TabaresPaul W. ElsinghorstFélix A. UrraPaula AlvitoFélix CarvalhoPaula DoFengjui ChenPaula JorgeFeng-Yih YuPaulo ValeFerenc BagiPavel BabicaFernanda Gobbi AmorimPavel V. AvdoninFernanda PortaroPaweł GlibowskiFernando GómezPaweł KubicaFilippo RossiPawel KwiatkowskiFiorella TonelloPaweł MarciniakFlaminia VincentiPedro Infante-CossioFlavia GirolamiPedro Reis-CostaFlorian HennickePeng LiaoFlorian SchröperPeramaiyan RajendranFrancesca FerreraPeta HarveyFrancesca MalvanoPeter A. KeyelFrancesco ChiesaPeter AmpimFrancesco GiansantiPeter LittleFrancesco Maria RestivoPeter MantleFrancesco Paolo FanizziPeter ParsonsFrancesco TiniPetr HenebergFrancesco VoltaPetr KarlovskyFrancisc BodaPhilippe GuerreFrancisco CruzPhilippe PintonFrancisco Espejo AlcaidePia Lopez-JornetFrank EssmannPierre DenysFrédéric DucancelPieter-Jan D. GunsFrederic LibersatPilar Vila-DonatFrederick J. KaskelPiotr DobrowolskiFredrik UhlinPiotr IwaniukFuchang LiPiotr Jan JedziniakFulvio CiriacoPiotr MorasiewiczFurong TianPiya TemviriyanukulGabriela Cobo JaramilloPo Teen LimGabriele Angelo VassalloPo-Hsuan LuGabriele BalciunaitePrabodh SatyalGajdoš Kljusurić JasenkaPradeep KumarGanesh BavikattePranshu SahgalGarbiñe OlabarríaPrashant JainGareth J. SangerPredrag CudicGary M. BucciarelliPriscila Tessmer ScaglioniGautam MehtaPriyanka ReddyGautam SethiPrzemysław GrelaGayane MartirosianPurba NagGeoffrey MasuyerPushpa Raj JoshiGeorge Cosmin NadǎşQiushui HeGeorge William KajjumbaQiuyun DaiGeorgia-Eirini DeligiannidouQi-Yin ChenGerald B. KoudelkaRaad NashmiGerald CohenRadhika AmaradhiGerald SchwerdtRafael PATINO-NAVARRETEGérard LizardRafal OgorekGerardo CorzoRaffaele CapassoGerd Fabian VolkRaffaele SerraGermán EbenspergerRaffaella RebucciGéza B. SelmeczyRaj KumarGiancarlo PerroneRajan AdhikariGihyun LeeRajiv ReebyeGina FerrazzanoRajtilak MajumdarGiorgio Lo GiudiceRam Manohar BasnetGiovanna FranciosaRamon AsisGiovanni GalassoRamon JaimeGiovanni LucaRamona KuhnGiovanni PalleschiRao L. DiviGirolamo CirrincioneRaquel OliveiraGiulio SeveriRavi VattepuGiuseppe BrisindaRay NortonGiuseppe Eros Massimino CocuzzaRaymond Scott DuncanGodefroid CharbonRebecca J. WhelanGodfrey WokorachRebecca LalchanGonçalo S. DuarteRebecca PooleGoo-Hyun MunRebecca TarvinGopinath MuruganandamReena KarthaGoran GajskiRegiane Rodrigues Dos SantosGoran KišReilly R. KayserGrazia GrecoReinhard WürznerGregorio VonoRenata VadkertiovaGregory CaputoRene KoeffelGriet GlorieuxReza Alizadeh KordabadGrzegorz OnikRicardo BessinGrzegorz WegrzynRicardo González-RezaGudula SchmidtRichard A. MandervilleGuillaume BlanchetRichard De BoerGuillermo MoreirasRichard DucatelleGurudeeban SelvarajRichard HeeremaGuy RostokerRichard LewisGwenaëlle Lavison-BompardRichard ProctorHae Woong ChoiRiikka PeltomaaHae-Jin LeeRita ChiaramonteHai YuRita PachecoHaiyuan CaiRita Restano CassuliniHana StarobovaRobert H. PoppengaHana ŠtěpánováRobert JarretHans J. BaeldeRobert KosickiHaquima El MourabitRobert KupczyńskiHarley L. WorthyRoberta BarrassoHarpal SinghRobin Lewis CooperHatzianestis IoannisRobin SuHazel FarrellRocío Ruiz LazaHeather FinlaysonRoderich SüßmuthHeather J. RayRoderick MackieHedvig FébelRoland BückerHee-Soo ParkRoland KlassenHeidi Udnes AamotRolf TeschkeHeike HermannsRoman PavelaHeinrich Schulte-BauklohRoman TürkHelena OstolazaRongsheng JinHelena SafaviRosa Durán-PatrónHema Prasad NarraRosanna CapparelliHeng ZhaoRoss DarnellHenricus A.M. MutsaersRudolf LucasHerbert Ryan MariniRui ChenHerbert SchmidtRui FalachoHermann-josef RothkoetterRussolina ZingaliHidetoshi InagakiRuta MucenieceHillary L. MehlRyuji KajiHiroki KimuraSabine PellettHiroki ShibataSafa OufensouHiroshi WatanabeSaid OulghaziHiroyuki YamadaSalvatore Giovanni De SimoneHisashi NishiwakiSamar IssaHolger BarthSamira AiliHorst PosthausSamuel D. RobinsonHouda BerradaSamuel E. AdunyahHrvoje BudinčevićSamuel EspinoHsin-I ChiangSamuel Ken-En GanHsin-Tang LinSándor Kunsági-MátéHuan ZhangSandra AguiarHue Jung ParkSandra BanackHui Wei FanSandra SousaHui-Seung KangSang Sun KangHuixiong LvSanjay GuptaHung HongSanjucta DuttaHung N. MaiSara CunhaHyang Sook ChunSara Duarte-SilvaHye Suk LeeSara García-LinaresHyun Jin ShinSara Mayo-PrietoHyunggoo KangSara TombelliHyung-Jin LeeSarah BigotHyun-Woo ParkSarah DoddIchiei NaritaSaran LotfollahzadehIda Sigueko Sano MartinsSari RämöIghli Di BariSascha RohnIgor SlivacSatoshi NagaiI-Hsiu HuangSatoshi WadaIlaria D’AcquaricaSaumik BasuIlaria GuerrieroSchyler A. EllsworthIlaria PelusoScott HughesIldikó Bata-VidácsSebastian GnatIlona SadokSebastian GornikIman Mehdizadeh GohariSebastián MasImourana Alassane-KpembiSebastian UlrichImrich BarákSébastien LarréchéIndre Kučinskaite-KodzeSebastjan BevcIne Van Der FelsSeiji InoueInês CarqueijeiroSelma SniniInese CakstinaSergey KozlovInga Sörensen-ZenderSergio Daishi SasakiIngars ReinholdsSergio Gómez-GrañaInho JoSergio MinucciInn-Ho TsaiSérgio SilvaIoanna StylianakiSeth M. DalyIoannis A. GiantsisShahid Ali RajputIoannis A. TsakmakidisShakir HasanIoannis SemposShan SuIoannis VamvakarisShauna Farr-JonesIosif MarincuSheng LiIria González-MariñoShiang-Jong TzengIrina SmeuShigeru SatoI-S. Wade HuangShihui LiuIsaac ThomsenShin TaketaIulian Alexandru GrosuShinichi IkushiroIva SovadinováShinji KishimotoIvan AbičićShinya SatoIvan PecorelliShirin AhmadiIvana Savic GajicShiro OkumuraIvica MatakShivam Om MittalIvo FridolinShoichiro SudaIvona LhotskáShrestha Sinha RayIwona GabrielShu WakinoIzabela ZakrockaShuen-Ei ChenIzwan BharudinShuowei CaiJ. David MillerSi-Cai ZhangJacek WąsowskiSijun ZhengJacob GalanSilke R. KleeJacqueline WiesnerSilverio RotondiJakub TomanSilvia GratzJames Henry DiazSilvia M. MihailaJames L. KlotzSilvina L. AriasJames MetcalfSimon SchiwekJan AfsetSimon Wade BaxterJan BobekSimona BuelliJan LubawySimona KavaliauskieneJan ZollSimona Marianna SanzaniJana MašlankováSimona PrencipeJana NačeradskáSimone BacchiocchiJanja BabičSimone VettorettiJan-Peter HildebrandtSiro LuvisettoJasmine YanSlawomir GonkowskiJason CarereSobhy El-SohaimyJason MagnusonSofia AgriopoulouJason PaxmanSofia CostaJason StricklandSoledad Pizarro-SánchezJassy DrakulicSolomiia KozachokJaume TorresSom S. ChatterjeeJavier UrracaSonghai TianJay W. FoxSonia MarinJean Pierre DescySonia Marin-SillueJean SavoieSonja DjudjajJean-Denis BaillySoo-Hyun SungJean-Luc RollandSophia BarinovaJean-Michel SavoieSophie LorberJeannette KluessSophie NdawJelena KruljSophie NicoleJelka PleadinSorina RopciucJennifer MitchellSouichi NakashimaJennifer MytychSpiridon MantzoukasJennifer R. McCallSpundana MallaJens FrisvadStacie C. SummersJer-Ming ChangStefan AsamJessica Dal ColStefan KahlertJessica M. LohmarStefania PollastroJesús Cosín-RogerStefanie GierJesús M. González-JartínStefano RavaioliJesús Morón-LópezStefano SuperchiJeyamalar JeyanathanStepan DenisovJhansi L. LeslieStéphane BurteyJiangeng HuangStéphane PoitevinJianlong LouStephane RetyJianmei YuStephen BeverleyJianping XuStephen DeyrupJian-Yong WuStephen MelvilleJie ChengSteve KiblerJie ZhengSteve PeigneurJih-Tay HsuSteven D. ColquhounJihwan MyungSteven T. HallerJin Joo ChaStoicho StoevJintae LeeSu Hyuk KoJi-won RyuSubba Rao ChagantiJoana PaulinoSudeb SahaJoana PrataSudharsan SadhasivamJoanna CzarnotaSung-Yong HongJoanna GajewskaSunil GairolaJoanna GruszczyńskaSunoh KwonJoão Carlos Caetano SimõesSusan P. McCormickJoão Guilherme CostaSusana LoránJoaquin Gomis-CebollaSuzanne HendrichJochen G. RaimannSvetlana SushkovaJoe M. O’SullivanSzilamer FerencziJoel Castro KraftchenkoSzymon BocianJoel SiegelTakahiro SakaiJoerg BlessmannTakahito ChijiwaJohan LedinTakako OsakiJohannes WöstemeyerTamas KomivesJohn DunbarTarek Mohamed Abd El-AzizJohn F. LeslieTariq MukhtarJohn KapolosTatiana S. FrolovaJohn-Ih LeeTeng PoyunJolanta WawrzyniakTimur Yu. MagarlamovJonathan McMurryTomas GirbesJordi MolgóTomasz W. KaminskiJose CaaveiroToshimitsu OndukaJosé Carlos MartinsTünde PusztahelyiJose GutierrezVerhaegen BavoJosé Javier Fernández CastroVictor M. Magdaleno-MadrigalJosé Luis José Luis García-CoronaVikash KumarJosé M. FerrerasVili DragomirJosé Rafael De AlmeidaVincenzo Li MarziJose Ramon PinedaViorica Railean-PlugaruJosef ElsterVladimir OstrýJosefa TolosaWei-Ning LinJoselito P. QuirinoWen-Chieh SungJoseph BartgesWenxiao JiangJosephine WeeWilliam J. MeggsJosette PerrierWilliam J. PlaczekJosette RaymondWilliam KemJoshua GibsonXiangping ChuJosué J. Da SilvaXin ChengJouko RikkinenXingmin SunJoyce TengXuexiang HeJR-LIN LINY. Andi TrisyonoJuan BlancoYang LiJuan BlasiYanshen LiJuan Francisco MartínYazine MahjoubJuan Manuel Germán-AcacioYi-Ting LinJuan R. Muñoz-CastañedaYoshizo MatsukaJuan RubioloYou-Jin ChoiJuan-García AnaYuji OkaJuan-Miguel Fregeneda-GrandesYuliya KorolkovaJudit MorelloYury BelyiJuho HautsaloYuseok MoonJuliana Pavan ZulianiYves HenryJuliana ScianiZdeněk FarkaJulien BarbierZhenjun SunJulien MasquelierZhi-Min HaoJun HamazakiZuzana Hruska


